# Leveraging generative AI to simulate mental healthcare access and utilization

**DOI:** 10.3389/frhs.2025.1654106

**Published:** 2025-08-26

**Authors:** Cortney VanHook, Daniel Abusuampeh, Jordan Pollard

**Affiliations:** ^1^ School of Social Work, University of Illinois at Urbana-Champaign, Urbana, IL, United States; ^2^ School of Social Work, University of Pittsburgh, Pittsburgh, PA, United States; ^3^ School of Human Services, University of Cincinnati, Cincinnati, OH, United States

**Keywords:** generative AI simulations, precision mental health care, access to care, measurement-based care, cultural competence

## Abstract

**Purpose:**

This article examines how generative artificial intelligence (AI) can simulate, analyze, and enhance mental health care journeys for individuals from diverse backgrounds, supporting improved access, personalization, and outcomes.

**Design/methodology/approach:**

An AI-generated case study of Marcus Johnson, a 24-year-old Black software developer in Atlanta, models the interplay of personal, cultural, and systemic factors affecting mental health care access. The analysis integrates Andersen's Behavioral Model, Penchansky and Thomas's Dimensions of Access, and Measurement Based Care (MBC) to systematically identify barriers, facilitators, and opportunities for data-driven intervention and tailored care.

**Findings:**

The case study demonstrates that generative AI simulations, especially when combined with MBC, can replicate real-world complexities, inform clinical decision-making, and personalize interventions through ongoing assessment, symptom monitoring, and collaborative planning. Telehealth, flexible scheduling, and cultural competence are highlighted as critical for bridging access gaps and improving outcomes.

**Originality/value:**

This work is among the first to synthesize leading access-to-care models, MBC, and generative AI to simulate and improve mental health care pathways. The approach offers a novel framework for educators, clinicians, and system designers to address the full spectrum of access challenges and clinical needs in contemporary populations.

**Practical implications:**

Generative AI, anchored in evidence-based frameworks, enables mental health professionals and trainees to test and refine care strategies in a risk-free environment, promoting more equitable, responsive, and effective mental health systems for all.

## Introduction

Generative AI technology is revolutionizing how mental health professionals' model and analyze healthcare access, particularly in complex, real-world scenarios. By inputting key client characteristics (e.g., demographic information, insurance status, cultural background, and health needs) mental health professionals can leverage AI to generate diverse simulations (1), forecast potential obstacles (2) and identify optimal intervention points (3). This scenario-building capability empowers practitioners to virtually test approaches before implementation, allowing for more precise and impactful strategies (1, 4). Rather than replacing clinical judgment, AI enhances it, offering a robust tool for addressing the multifaceted challenges of healthcare access.

This article demonstrates the potential of generative AI in mental health by simulating the care journey of Marcus Johnson, a young Black man navigating Atlanta's mental health system. Leveraging Andersen's Behavioral Model, we show how AI can dynamically replicate the interplay of predisposing, enabling, and need factors that shape patient experiences (5). In addition, we utilize the Penchansky and Thomas Dimensions of Access (6) to systematically evaluate how simulated interventions address barriers such as availability, accessibility, affordability, acceptability, and accommodation within the mental health care system. We also explore the integration of Measurement Based Care (MBC), an evidence-based model, within these simulations to refine intervention strategies (7). Together, these approaches illustrate how generative AI, combined with established frameworks, can transform our understanding and improvement of mental health care pathways.

This study explores whether generative AI, when coupled with established theoretical frameworks, can effectively simulate and illuminate the complex factors of mental health access for a young Black man. This case study will leverage Andersen's Behavioral Model, the Penchansky and Thomas Dimensions of Access, and Measurement-Based Care (MBC) principles to analyze barriers and opportunities for intervention. We hope that the framework of this study will contribute to both clinical practice and training in mental health professions (e.g., social work, psychology, marriage and family therapy, psychiatry, psychiatric nursing, etc.).

## Methods

### Rationale for simulation and ethical considerations

We chose an AI-generated simulation case (rather than a real or composite clinical case) to construct a prototypical, de-identified client journey that encapsulates common access barriers while protecting privacy. In addition, AI-generated simulations offer notable time efficiency compared to recruiting, de-identifying, and synthesizing real or composite clinical cases. This allows educators and researchers to rapidly create diverse, contextually relevant scenarios tailored to specific learning objectives or research questions, making simulation-based training and inquiry more accessible and scalable. The simulation draws on empirical data and clinical experience, guided by demographic, cultural, and psychological patterns in literature. Generative AI is likely to be increasingly used to create clinical case studies for education and research as these technologies mature. We acknowledge limitations, including reduced unpredictability and emotional nuance, and potential risks related to authenticity and representation.

### Simulation design and methodological transparency

The simulation was generated using HyperWrite (39), which utilizes OpenAI's GPT-4 large language model (LLM) for text generation. GPT-4 is a neural network-based model trained on extensive datasets that include clinical literature, academic sources, and diverse real-world text. HyperWrite's text generation process synthesizes information and produces clinically realistic scenarios by drawing on its underlying LLM's ability to recognize patterns, contextual nuance, and evidence-based best practices. For this study, HyperWrite was prompted with a detailed, structured case brief outlining Marcus's demographics, symptoms, psychosocial context, and insurance status, all grounded in published research on mental health barriers for Black men in the U.S.

**Figure 1:**
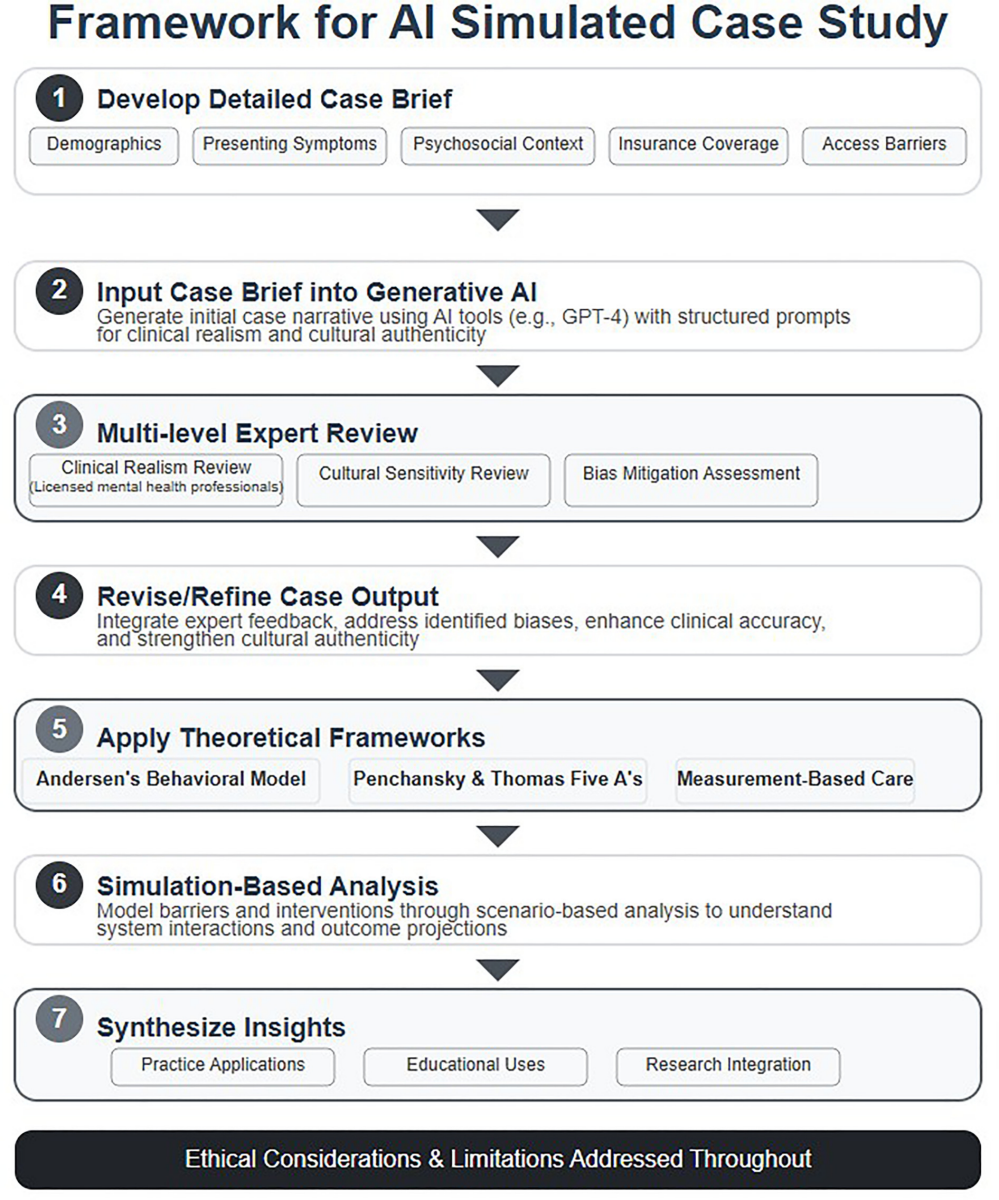
Framework for AI simulated case study.

To ensure that the AI-generated simulation reflects real-world clinical practice, the case brief and subsequent outputs were compared against published research and on barriers faced by Black men in the U.S. mental health system [e.g., (8, 9)]. All AI outputs were reviewed and edited by licensed mental health professionals (authors CV and JP) to ensure clinical accuracy, cultural sensitivity (all three authors identify as Black men), and alignment with current standards for case formulation. Outputs were revised or omitted if they risked oversimplification or misrepresentation. This triangulation increases the validity of the simulation and addresses concerns about the inherent limitations of AI-generated data. See Figure 1 for the framework of this AI simulated case study.

### Theoretical frameworks for analysis

Andersen's Behavioral Model (10) is a foundational framework for understanding why individuals use health services, positing that healthcare utilization is determined by the interplay of three categories of factors: predisposing characteristics (such as demographics and health beliefs), enabling resources (like insurance and provider availability), and need factors (both perceived and professionally evaluated). By considering these components, the model provides a comprehensive lens through which to analyze the multifaceted barriers and facilitators shaping access to care, making it especially valuable for examining disparities and tailoring interventions to improve health service uptake across diverse populations (5).

The Penchansky and Thomas model, often referred to as the “Five A's” framework, conceptualizes access to healthcare as the degree of fit between patients and the health system across five distinct dimensions: availability, accessibility, accommodation, affordability, and acceptability (11). Each dimension represents a unique aspect of the interaction between individuals and care providers, emphasizing that barriers to access can arise from mismatches in service supply, organization, location, cost, or cultural alignment (6). This model offers a practical lens for diagnosing gaps in healthcare delivery and has become especially influential for evaluating and improving real-world access to care.

Measurement Based Care (MBC) is an evidence-based approach that involves the systematic use of standardized assessment tools to track patient symptoms, monitor treatment progress, and inform clinical decision-making in real time (12). By regularly collecting and reviewing objective data (e.g., depression or anxiety scores) providers can tailor interventions to individual needs, promptly identify when care adjustments are necessary, and actively engage patients in their own treatment plans (12). MBC moves beyond subjective impressions, promoting transparency, collaboration, and improved outcomes in mental health care (12). MBC has been shown to enhance patient satisfaction, detect failure to respond to treatment, decrease likelihood of deterioration, improve treatment adherence, reduce the need for emergency services, and accelerate recovery [see reviews by (12–14)].

## Results: simulation case study—Marcus Johnson

### Artificial AI prompt

Generate a detailed clinical case study for Marcus Johnson, a 24-year-old single Black man in Atlanta who works as a software developer. He has no prior mental health treatment, is physically healthy, and presents with six months of low mood, fatigue, insomnia, loss of interest, irritability, and difficulty concentrating after a breakup and increased job stress. He denies suicidal ideation and substance use. Raised in a supportive, achievement-oriented Black family, Marcus is close to his mother (supportive and nonjudgmental), has a father who encourages him (but with whom he's not comfortable discussing mental health), and a female cousin open about her own mental health struggles, which has helped Marcus's address stigma. He's withdrawn from friends and activities, is concerned about mental health stigma, and faces barriers including lack of Black male therapists and concerns about cultural competence. Please include presenting problem, history, social/family context, mental status, protective factors, barriers to care, diagnosis (major depressive disorder, moderate), and a culturally responsive, measurement-based treatment plan. Present the case in a clear, concise clinical format.

### Descriptive case study: Marcus Johnson

#### Identifying information

Marcus Johnson is a 24-year-old, single, cisgender Black man living in Atlanta, Georgia. He was born and raised in Atlanta and is the first in his immediate family to obtain a college degree. He currently works as a full-time software developer at a mid-sized technology firm.

#### Referral source and reason for referral

Marcus was encouraged to seek counseling after attending a workplace wellness seminar highlighting mental health resources available through his employer's Employee Assistance Program (EAP). He self-referred for services, reporting concerns about mood and work performance.

#### Presenting problem

Marcus reports a six-month history of persistent low mood, diminished interest in previously enjoyable activities (e.g., basketball, gaming with friends), fatigue, and difficulty concentrating. He notes increased irritability, social withdrawal, and frequent insomnia characterized by early morning awakening. Marcus describes feeling “disconnected” and “unmotivated” both at work and at home. He denies any suicidal ideation, homicidal thoughts, or psychotic symptoms.

#### History of present illness

These symptoms gradually developed over several months following a failed romantic relationship and increased pressures at work due to a major project deadline. Marcus initially attributed his difficulties to temporary stress but has grown increasingly concerned as his symptoms have persisted and intensified, interfering with his job performance and daily functioning.

#### Past psychiatric & substance use history

Marcus has no prior history of formal mental health treatment, psychiatric hospitalization, or use of psychotropic medication, though he recalls occasional “down periods” during college, none as prolonged or impairing as his current episode. He drinks alcohol socially (1–2 drinks, 1–2 times per month) and denies any tobacco or illicit drug use.

#### Medical history & family history

Marcus is in good general health, with a history of mild asthma managed with an inhaler as needed and no past surgeries or chronic illnesses; he does not take any regular medications aside from his inhaler. Family Psychiatric and Medical History: There is no known family history of mood disorders, psychosis, or substance use disorders; his mother has hypertension, his father is generally healthy, and there is no reported family history of suicide.

#### Developmental and social history

Marcus grew up in a close-knit family in a predominantly Black middle-class neighborhood. He describes his upbringing as supportive but notes that his family culture emphasized self-reliance and emotional stoicism (“handle your business”). Marcus excelled academically and was active in high school sports. He maintains regular contact with his family but has noticed decreased communication recently due to his low mood.

#### Occupational/educational history

Marcus graduated with honors from a state university and quickly found employment as a software developer. He has been with his current employer for two years and generally receives positive performance evaluations. Recently, he has struggled to meet deadlines and feels less confident in his work.

#### Relationships and support system

Marcus is single and lives alone, and while he has close friends, he's been withdrawing, feeling “out of sync.” Despite this isolation, he's close to his supportive mother, who provides a nonjudgmental space for him. He also considers his father part of his support system; his father offers motivation and encouragement, though Marcus isn't comfortable discussing mental health with him. A cousin's openness about her own mental health challenges has helped normalize these conversations and reduce stigma for Marcus. His mother's support, his cousin's openness, and his father's encouragement are a crucial foundation as he navigates his difficulties.

#### Cultural/spiritual identity

Marcus identifies strongly with his Black heritage and has a positive sense of cultural pride. He was raised in a Baptist church but currently attends only occasionally. He describes his faith as a “quiet source of strength” but has not actively sought support from his church community.

#### Mental status examination

Appearance: Marcus presented as well-groomed and casually dressed. Behavior: He was cooperative and reserved, maintaining eye contact throughout the evaluation. Speech: His speech was normal in rate and tone. Mood: He described his mood as “low” and “unmotivated.” Affect: His affect was restricted in range, congruent with his reported mood. Thought Process: His thought process was logical and goal directed. Thought Content: He denied any delusions, suicidal ideation (SI), or homicidal ideation (HI). Perceptions: He denied any hallucinations. Cognition: He was alert and oriented to person, place, and time (×3), with mild difficulty with attention and concentration. Insight/Judgment: His insight and judgment were good.

#### Risk assessment

Marcus denies suicidal or homicidal ideation, intent, or plan. No history of self-harm or violence. Protective factors include strong family ties, employment, and future orientation. Marcus's strengths include his high educational attainment, strong work ethic, insight into his symptoms, willingness to seek help, supportive family background, and access to employer-sponsored mental health resources. Several barriers could impede Marcus's access to care, including the stigma around mental health in his community, the lack of Black male therapists in-network, concerns about provider cultural competence, and apprehension about missing work for appointments.

#### Initial clinical formulation and diagnosis

Marcus's presentation is consistent with a first episode of Major Depressive Disorder, moderate, single episode, without psychotic features (DSM-5-TR criteria). His symptoms are significantly impacting occupational and social functioning. No evidence of substance misuse, psychosis, or mania.

#### Initial treatment plan

Initiate short-term, measurement-based psychotherapy (CBT or culturally adapted therapy). Virtual sessions should be scheduled through the EAP to accommodate his work schedule. Conduct PHQ-9 and GAD-7 assessments at intake and each session to monitor progress. Provide psychoeducation about depression and cultural considerations. Encourage gradual re-engagement in pleasurable and social activities. Reassess the need for a psychiatric evaluation if there is no improvement.

#### Prognosis

With early intervention, strong support systems, and ongoing measurement-based care, Marcus has a favorable prognosis. Key to his recovery will be culturally sensitive engagement and collaborative goal setting.

#### Cultural and ethical considerations

The case highlights the importance of addressing stigma, cultural mistrust, and provider diversity in mental health care for Black men. The AI-generated nature of this case means it serves as a composite scenario meant for educational purposes and should not substitute for direct patient care or lived experience.

### Application of theoretical frameworks

#### Application of Andersen's behavioral model to Marcus Johnson

**Figure 2:**
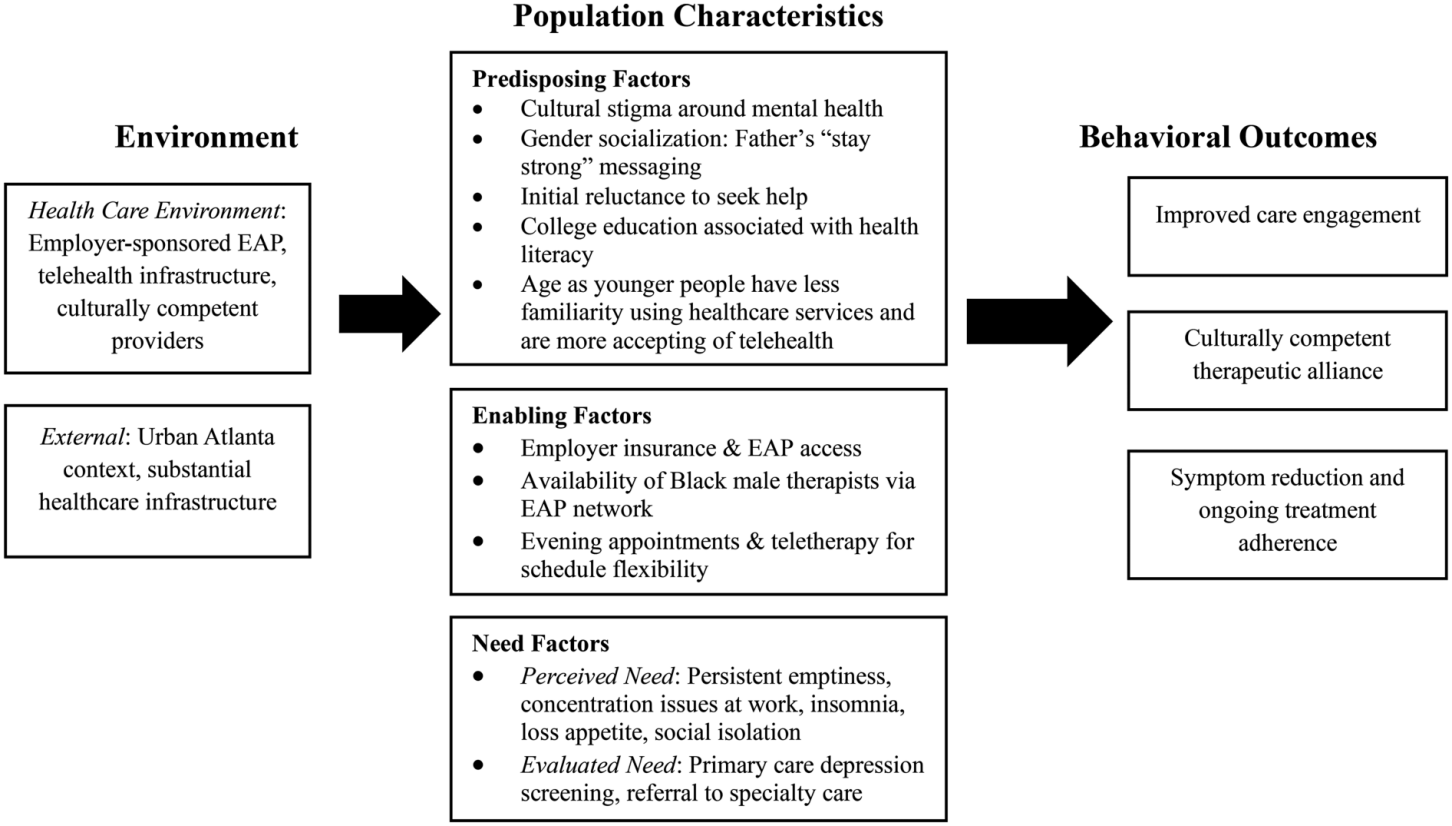
Andersen's behavioral model—marcus edition (author's property).

The application of Andersen's Behavioral Model (Figure 2) explains health service use through predisposing, enabling, and need factors (5). In Marcus's case, *predisposing factors* include his identity as a young, Black, college educated man and the family value of self-reliance, both of which contribute to internalized stigma and initial reluctance to seek care (15, 16). *Enabling factors* such as employer sponsored insurance, access to an EAP, and the presence of supportive family members help facilitate his care, though persistent barriers like the shortage of culturally competent providers and work schedule constraints remain relevant (8, 9). Marcus's emerging recognition of his symptoms and his family's concern illustrate how perceived *need* can become a decisive factor in seeking help (17). His progression from hesitation to engagement highlights the model's interplay of factors, as supportive resources and increasing need begin to outweigh cultural and systemic barriers.

#### Marcus Penchansky and Thomas's dimensions

**Figure 3:**
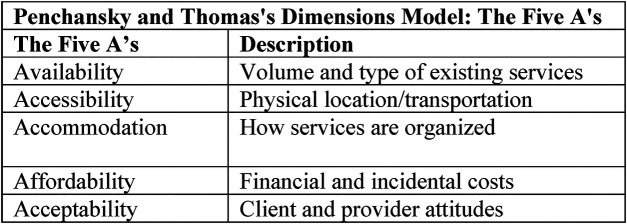
Penchansky and Thomas 5 dimensions (author's property).

Analyzed through the Five A's framework, Marcus's experience demonstrates both gaps and successes in healthcare access (Figure 3). *Availability* is challenged by the limited number of Black male therapists (18) within his EAP network, but persistence and the option of virtual care eventually resolve this barrier. *Accessibility* is enhanced by telehealth, allowing Marcus to overcome logistical hurdles posed by Atlanta's geography and his busy work schedule (19). *Accommodation* is achieved through flexible scheduling, such as evening teletherapy, directly addressing his work-related constraints. *Affordability* is supported by insurance and EAP benefits, minimizing out of pocket costs for Marcus. *Acceptability* increases as Marcus's treatment aligns with his cultural expectations, aided by family support and a culturally competent provider (8, 20). This analysis underscores how modern delivery models, when thoughtfully implemented, can address longstanding access barriers for Black men.

#### Measurement based care integration

**Figure 4:**
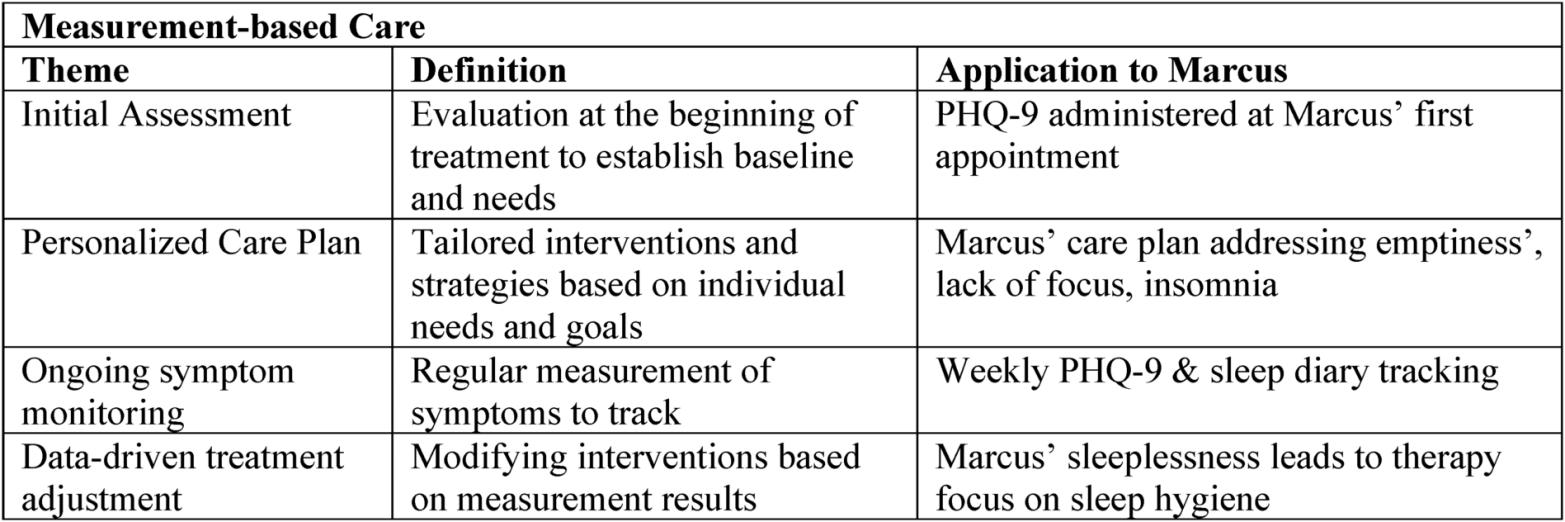
Measurement-based care—Marcus edition (author's property).

At the outset of care, Marcus completes standardized tools (PHQ 9, GAD 7) to establish a quantitative baseline for depression and anxiety *(initial assessment)* (21, 22). These results, combined with discussion of Marcus's goals and cultural context, inform a *personalized care plan* that includes preferences for provider characteristics, flexible appointments, and culturally sensitive strategies (23–25). *Ongoing symptom monitoring* is achieved through these measures at each session, providing timely data for Marcus and his provider to track improvement or deterioration (26–28). This regular tracking enables *data driven treatment adjustment*—if scores improve, the plan is maintained; if not, barriers are reviewed and strategies are adapted, which may involve changes in therapy, referral for medication, or addressing nonclinical barriers (29, 30). Throughout, *feedback and shared decision making* are central: Marcus's provider reviews results with him using clear, jargon free language and encourages input, ensuring the care process adapts to his needs and cultural context (31–34). This collaborative approach increases Marcus's engagement, supports the therapeutic alliance, and fosters sustained positive outcomes (Figure 4).

## Discussion

Generative AI is poised to transform mental health clinical practice by providing clinicians with advanced tools for understanding and addressing complex behavioral health challenges. Through realistic simulations and adaptive technologies, AI enables providers to analyze the personal, cultural, and systemic factors that influence patient care, supporting the field's ongoing movement toward greater equity and cultural competence (35, 36). The case of Marcus Johnson demonstrates how AI driven scenarios, particularly when integrated with Measurement Based Care, facilitate data driven monitoring of patient progress, more collaborative treatment planning, and timely adjustments to interventions. This approach allows for the individualization of care, enhances cultural sensitivity, and helps optimize outcomes thereby contributing to a more responsive and effective mental health system (37, 40). This presents new opportunities to enhance patient care through tailored treatment plans and culturally sensitive interventions.

In addition to its clinical applications, this article offers a flexible structure for education and training across the mental health disciplines. As AI becomes increasingly incorporated into college assignments and curricula, this model provides a practical template for combining AI based case simulation, treatment planning, and the development of clinical judgment (38). Students can interact with culturally nuanced, realistic case material through course papers, classroom presentations, and as a foundation for theses or dissertations. The methodology can also be integrated into field placements and internships, helping students adapt to new populations and unfamiliar clinical scenarios (38). Incorporating AI driven simulations into these educational settings not only facilitates experiential learning but also prepares future practitioners for the evolving complexities of real-world mental health care.

While this study demonstrates the promise of generative AI in simulating complex mental health scenarios, several limitations must be acknowledged. The AI-generated case is limited by the data and patterns in the model's training set, which may not reflect the full diversity, unpredictability, or emotional nuance of real clinical encounters. Such simulations cannot substitute for lived experience or capture every cultural, social, or individual factor influencing care. Despite expert review to enhance accuracy and cultural sensitivity, outputs remain subject to bias and oversimplification. The frameworks applied, though evidence-based, do not address all systemic and structural barriers Black men face. As AI evolves, ongoing evaluation and transparency will be essential for responsible and equitable use in education and practice. Future research should compare AI-based simulations with real-world cases and consider additional frameworks, such as intersectionality or structural competency, to better capture the full spectrum of social determinants and systemic barriers to care.

## Conclusion

Generative AI offers a novel approach for both clinical practice and training by enabling the rapid creation of complex, culturally relevant case studies. These simulations bridge the gap between theory and practical application, allowing clinicians and students to analyze barriers to care, test intervention strategies, and refine treatment planning in a controlled, risk-free environment. As mental health care continues to evolve, integrating AI into practice and education holds significant promise for improving access, cultural competence, and patient outcomes.
